# Integration of 3D Structure from Disparity into Biological Motion Perception Independent of Depth Awareness

**DOI:** 10.1371/journal.pone.0089238

**Published:** 2014-02-21

**Authors:** Ying Wang, Yi Jiang

**Affiliations:** State Key Laboratory of Brain and Cognitive Science, Institute of Psychology, Chinese Academy of Sciences, Beijing, China; Centre de Neuroscience Cognitive, France

## Abstract

Images projected onto the retinas of our two eyes come from slightly different directions in the real world, constituting binocular disparity that serves as an important source for depth perception - the ability to see the world in three dimensions. It remains unclear whether the integration of disparity cues into visual perception depends on the conscious representation of stereoscopic depth. Here we report evidence that, even without inducing discernible perceptual representations, the disparity-defined depth information could still modulate the visual processing of 3D objects in depth-irrelevant aspects. Specifically, observers who could not discriminate disparity-defined in-depth facing orientations of biological motions (i.e., approaching vs. receding) due to an excessive perceptual bias nevertheless exhibited a robust perceptual asymmetry in response to the indistinguishable facing orientations, similar to those who could consciously discriminate such 3D information. These results clearly demonstrate that the visual processing of biological motion engages the disparity cues independent of observers’ depth awareness. The extraction and utilization of binocular depth signals thus can be dissociable from the conscious representation of 3D structure in high-level visual perception.

## Introduction

Perceiving the world in three dimensions is vital for our survival and daily activities. Without seeing depth, we would not be able to tell how far away a lion in the wild is from us or to intercept a flying ball. Fortunately, the human visual system possesses a remarkable capability to reconstruct the 3D world from flattened retinal images based on a variety of depth cues. For example, with binocular disparity, the difference of retinal images resulting from the horizontal separation of the two eyes, our brain is able to extract stereoscopic depth with excellent precision [1]. On the other hand, depth perception is highly susceptible to top-down regulation due to its constructive nature, and the neural resolution of depth cues can be overrode and even fail to elicit a veridical perception of the 3D reality [2]. The most famous demonstration of this phenomenon is the hollow face illusion: the back of a facial mask is stunningly and robustly misinterpreted as a convex face rather than a concave mask [3], with the strength of the illusion enhanced by the familiarity of the stimulus property [4].

What is the cognitive fate of those perceptually suppressed depth information? Do they still engage in the visual processing of 3D objects? Despite the abundant evidence that disparity computation in the brain is a multi-stage process [1], [5], [6] with the explicit representation of stereoscopic depth accomplished beyond the primary visual cortex [7], [8], it remains unclear whether the disparity-related signals can be integrated into relatively higher stage of visual processing independent of the awareness of depth. It is also unknown whether the perceptually unresolved stereoscopic information can play a functional role in 3D object perception.

To investigate these issues, we adopted point-light walkers [9], an instantiation of biological motion that vividly simulates the movements of human figures. It has been demonstrated that the visual perception of point-light walkers is intrinsically 3D and supported by neural mechanisms that integrate form, motion, and depth information [10]. When projected orthographically on the screen, these stimuli do not provide definite information regarding their facing orientations in depth and thus give rise to bistable depth perception [11]. However, there is an overall yet observer-dependent tendency among normal population to perceive the ambiguous point-light walkers as facing toward the viewer (FTV) more often than facing away, i.e., a FTV bias [12]–[15], indicating a top-down influence on the depth perception of biological motion. Moreover, the top-down influences on the visual interpretation of 3D point-light walker may even overwhelm the depth information provided by the binocular disparity cues [2].

In the current study, we first reported a novel phenomenon that the stereoscopically defined in-depth orientation of point-light biological motion switched physically between two opposite orientations (i.e., approaching vs. receding), while the observer’s percept remained constant due to a strong top-down influence, i.e., the FTV bias. Such phenomenon provides a unique opportunity to examine the integration of disparity cues that are inaccessible to depth awareness into the perception of meaningful 3D objects. To examine the function of the perceptually unresolved depth cues, we tested the observers who could not discriminate the disparity-defined in-depth facing orientations (the experimental group) on biological motion perception tasks that were irrelevant to the depth property of the stimuli. If these observers exhibited a perceptual asymmetry arising from the disparity-defined in-depth facing orientation, similar to those who could accurately identify such information (the control group), it would suggest that disparity cues could be integrated with other visual cues in 3D biological motion perception independent of the subjective experience of stereoscopic depth.

## Materials and Methods

### Ethics Statement

The experimental procedures and protocols were approved by the institutional review board of Institute of Psychology, Chinese Academy of Sciences. All participants gave written, informed consent prior to testing.

### Participants

Thirty-one naïve observers and one author participated in the study (see Methods S1 for more information about the prescreening of participants). All took part in Experiment 1a and were assigned to the experimental (n = 14, mean age = 22.5 years, 7 males) or control (n = 18, mean age = 22.1 years, 7 males) group. All observers from the experimental group took part in Experiment 1b and 1c, 12 in Experiment 2a, 10 in Experiment 2b, and 10 in Experiment 3. Accordingly, 12 and 10 observers from the control group also participated in Experiment 2a and Experiment 3, respectively. All participants had normal or corrected-to-normal visual acuity.

### Apparatus and Stimuli

The experiments were programmed using MATLAB with the Psychophysics toolbox extensions [16], [17]. Point-light stimuli, rendered in white against a uniform grey background, were displayed on a 21-inch CRT monitor (1280×1024, 60 Hz). Observers viewed stimuli from a mirror stereoscope mounted on a chin rest with the viewing distance set to 60 cm. During all experiments, a square frame (16.5°×16.5° in visual angle) with a centered fixation cross was presented on both the left and right halves of the screen to facilitate binocular fusion.

The biological motion sequence, consisting of fifteen luminous dots located on the head and key joints of an animated walker, mimicked a person walking on a treadmill [18]. Each sequence contained one gait cycle lasting 1 second and was rendered smoothly at 30 frames/s. To create stereo walkers, a pair of biological motion sequences was displayed dichoptically using the mirror stereoscope. The retinal positions of the point-lights that portrayed the walker were set slightly different from the left to the right eye to manipulate the disparity information received by the observer, thus unambiguously defining the in-depth facing orientation of the walker, i.e., toward or away from the viewer (see **Animation S1 and Animation S2**).

The walker subtended 2.38°×8.44° in visual angle. The relative disparity between the nearest and the farthest points within the walker was 21 arcmin. Similarly, stereo sphere stimulus, composed of 100 dots on its virtual surface, was dichoptically presented and defined as either rotating toward or away from the viewer with reference to the summit. The speed of rotation was 60°/s. The sphere subtended 3.58° in both width and height. Relative disparity was about 16 arcmin between the nearest and the farthermost point.

### Procedure

In Experiment 1a, the stimuli were point-light walkers with two types of in-depth facing orientations (toward vs. away). Each trial started with a 1000 ms interval of stimulus presentation, followed by a response stage with no time limit. Observers were required to report, as accurately as possible, whether the stereo walker was facing toward or away from them by pressing one of two keys. To avoid the potential response bias linked with low-level features, the facing orientation of the walker was randomly deviated from the axis of depth within a small range (left 7.5°, 5°, 2.5°, right 2.5°, 5°, 7.5°, or 0°). The formal test block contained 70 trials. Before the formal test, we also conducted several practice blocks that applied the same procedure as the main test, except that feedback was provided after some trials, to ensure that the participants got familiar with the apparatus and stimuli. Experiment 1b adopted the same design as Experiment 1a except that the stimuli were point-light rotating spheres, and the task was to judge whether the sphere was rotating toward (front surface moving downward) or away from the observers (front surface moving upward). Experiment 1c employed a two-interval forced-choice paradigm. In each trial, two 1000 ms biological motion sequences, with either the same or opposite facing orientations in depth, were displayed successively with a randomized inter-stimulus interval from 400 to 600 ms. After the stimuli disappeared, the observers were required to judge whether the two walkers were the same or not. There were 56 trials, half for the same facing orientation (with equal number of toward and away trials) and the other half for the different facing orientations (with the sequential order balanced).

Experiment 2a had the same design and procedure as Experiment 1a except that 1) there were 140 trials with 10 in each facing direction condition (7 deviation angles, either toward or away), and 2) observers were required to judge whether the walking direction of the walker was deviated slightly to the left or right, regardless of its facing orientation in depth. Experiment 2b differed from Experiment 2a only in the test stimuli, i.e., the 3D point-light rotating spheres replaced the point-light walkers.

Experiment 3 employed a detection task. In each trial, an upright (target) or inverted (distractor) point-light walker was embedded in dynamic noise [19], and the observers’ task was to report whether the target was present or not. For each trial, the noise was created by overlapping five different scrambled versions of the intact walker, moving in either the same or opposite orientation through depth (congruent vs. incongruent) with regard to the test stimuli, and was distributed approximately 20 percent wider in horizontal and vertical dimensions than that covered by the test stimuli. There were totally 120 trials, with a combination of three fully balanced conditions: in-depth facing orientations (toward vs. away), target presence (present vs. absent), and disparity of noise (congruent vs. incongruent with the test stimuli).

In all experiments, a small random spatial displacement (0.01°∼0.02°) was added to the test stimuli in order to avoid potential visual interference from the previous trials. The initial frame of the motion sequences was also randomized for each trial. Trials of different conditions were mixed and presented in randomized order. The inter-trial intervals were set to 1000 ms.

### Data Analysis

To evaluate the sensitivity of left-right walking direction discrimination, responses of Experiments 2a and 2b were transformed into percentage of “right” responses (i.e., the walker was perceived as deviated to the right direction) for each deviation angle. The data were then fit with a Boltzmann sigmoid function (f(x) = 1/(1+exp ((x−x0)/ω))) for each in-depth facing orientation condition and for each individual observer. Discrimination threshold was measured as the angle distance between the points where observers were at 25 and 75 percent of chance to make “right” responses (i.e., the interquartile range of the fitted function). Lower threshold or smaller angle distance indicated higher sensitivity of left-right walking direction discrimination (see (Luce & Galanter, 1967) for a more detailed explanation).

## Results

### Experiment 1: Overwhelmed Stereopsis in Biological Motion Perception

**Figure 1:**
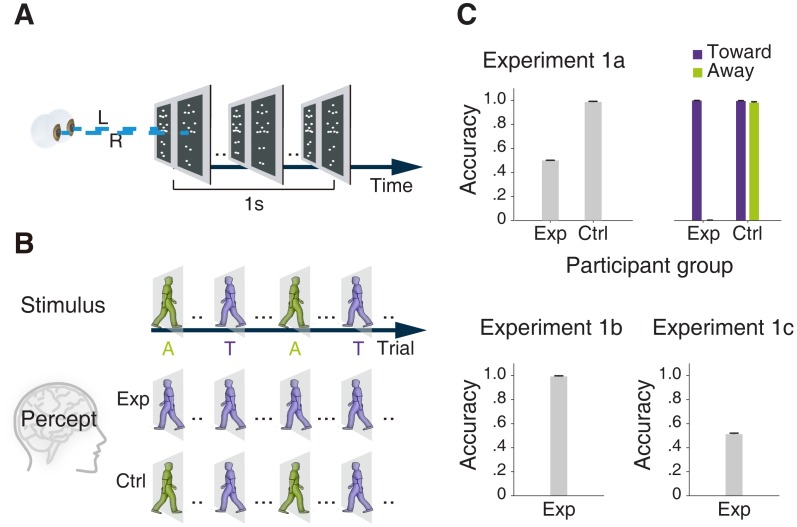
Schematic representations of 3D biological motion stimuli and results of Experiment 1. (A) During each trial, a pair of point-light walker sequences with horizontal binocular disparity was presented to the left and right eyes of the observer through a mirror stereoscope. (B) A series of point-light walkers, either facing toward (T-trial) or away (A-trial) from the observer defined by stereoscopic cues were displayed in random order. Observers were divided into experimental and control groups according to their perceived depth information. (C) The upper panel shows the contrast of accuracy for in-depth facing orientation judgment between the two groups, with the physical in-depth facing orientations combined or separated. Observers in the experimental group correctly identified the in-depth rotating direction of point-light sphere (lower left panel), yet their performance for discriminating the depth-reversed point-light walkers was at chance level (lower right panel). Error bars indicate one SEM.

In Experiment 1a, we identified two subgroups of observers with regard to their perceived in-depth facing orientations of stereoscopically presented point-light biological motions, whose depth information was defined by disparity cues (Figure 1). The experimental group contained those who constantly perceived the walkers as walking toward them and therefore performed only 50% correct (chance level) in the in-depth facing orientation judgment task. In contrast, the control group could perceive and identify the in-depth facing orientation with almost 100% accuracy. If we set apart the two in-depth facing orientation conditions, the experimental group showed accuracies of 100% and 0% for the facing toward and facing away conditions, respectively (*t* (13) = 359.16, *p*<0.001), while no such difference was observed for the control group (*t* (17) = 1.70, *p*>0.1). This pattern of results produces a significant interaction (*F* (1, 30) = 9891.00, *p*<0.001) between walker’s in-depth facing orientation (toward vs. away) and participant group (experimental vs. control).

We further tested the observers from the experimental group with two additional tasks. Results of experiment 1b showed that, all of the observers were able to correctly identify the in-depth orientation of stereo rotating spheres (either toward or away from the viewer) defined by binocular disparity cues in a way similar to that of the stereo walkers (Figure 1c). Moreover, in an additional experiment (see Methods S1), we found that these observers had normal stereoscopic depth discrimination threshold for random-dot stereograms (3.3 arcmin), which was equal to that observed from those in the control group (*t* (8) = 0.08, *p*>0.1). Therefore, their failure in judging the in-depth orientation of biological motion was neither due to abnormal stereoscopic sensitivity nor to a generic bias for structure-from-motion perception. Experiment 1c further examined the observers’ ability to discriminate depth-reversed 3D biological motion stimuli with a more objective measurement. Observers were required to judge whether two successively displayed walkers, with either the same or opposite depth orders but otherwise replicated each other, were the same or not based on whatever cues they might perceive. Results confirmed that the observers were no better than random guessing and their overall accuracy was at chance level (Figure 1c, one sample t-test, *t* (13) = 1.59, *p*>0.1). Moreover, the statistical analysis of the individual-level performance based on the accumulative binomial probability of random process suggests that no observer had more than 5% probability of seeing the true difference between the depth-reversed walkers [20]. In other words, they were unable to resolve, perceptually, the phenomenal differences indicated by reversed binocular disparity cues that specified the in-depth orientations of the biological motion signals.

### Role of Unperceived Stereoscopic Cues in Biological Motion Perception (Experiment 2 & 3)

We have so far demonstrated that binocular disparity, which disambiguates the stimuli’s depth property physically, may not elicit corresponding depth perception of biological motion. A more interesting question was whether such perceptually indistinguishable depth cues play a functional role in visual perception. Because the approaching action of biological agent enjoys an advantageous processing in visual perception [21], [22], we conjectured that disparity-defined in-depth facing orientations may modulate biological motion perception by sensitizing the observer to point-light walkers who are walking toward them as opposed to those walking away (i.e., a FTV perceptual advantage). More critically, if the utilization of disparity cues in visual perception does not rely on the explicit representation of depth information, such modulation should take effect independent of whether the stereoscopic information was perceptually distinguishable. In other words, we should observe similar modulation effect with observers from the experimental and the control groups. In order to test these hypotheses, we adopted a left-right walking direction discrimination task (Experiment 2) as well as a detection task (Experiment 3), which have been used to reveal distinct aspects of biological motion processing in the previous studies [23], [24].

### Experiment 2: Perceptually Indistinguishable Stereoscopic Information Contributes to Left-right Walking Direction Discrimination of Biological Motion

**Figure 2:**
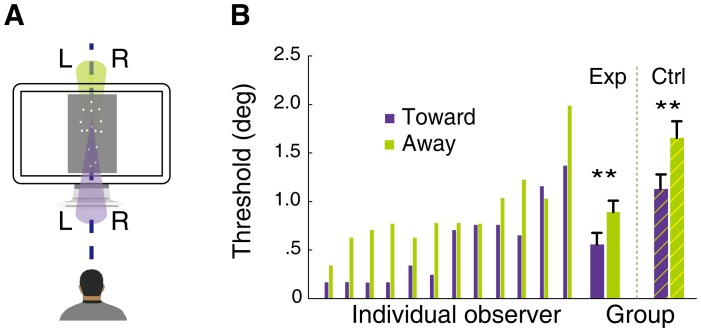
Experimental conditions and results of the left-right walking direction discrimination experiment. (A) Walking directions of stereo point-light walkers, defined by disparity cues (Purple - toward; Green - away), deviated slightly from the observer’s line of sight (L - Left; R - Right). (B) Left-right discrimination thresholds of toward and away conditions plotted for individual observers (the experimental group) and as group averages (the experimental and the control groups). The threshold of the toward condition was significantly lower compared with that of the away condition. Error bars indicate one SEM. **p<.01.

Figure 2 illustrated the experimental conditions and the results of Experiment 2a. As expected, disparity-defined orientations in depth induced a perceptual asymmetry effect. Particularly, performance was better (lower differential threshold) for 3D point-light walker presented as facing toward the viewer than facing away from the viewer (mixed-design ANOVA, *F* (1, 22) = 38.44, *p*<0.001). Most importantly, there was no interaction between in-depth orientation and participant group (*F* (1, 22) = 1.92, *p*>0.1). Observers in the experimental group who could not perceptually discriminate the in-depth facing orientations, similar to those in the control group, still showed a significant perceptual asymmetry effect (Experimental group: *t* (11) = −4.52, *p*<0.01; Control group: *t* (11) = −4.48, *p*<0.01). These results are in sharp contrast with those of Experiment 1a (depth perception), and suggest that information defined by binocular disparity can be extracted independent of depth awareness and integrated into visual perception to modulate the processing of the left-right deviation of the walking direction. We also noticed that the experimental group performed better overall than the control group in this discrimination task (*F* (1, 22) = 12.71, *p*<0.01), probably due to that the formers always perceived the same in-depth facing orientation and thus were less distracted by such task-irrelevant information. These findings together suggest that the extraction and utilization of disparity cues are rather automatic and independent of observers’ explicit attention to the depth information.

In Experiment 2b, we further examined whether the depth-related perceptual asymmetry reflected a general effect that can be extended to non-biological movements. We applied the same motion direction discrimination task to stereo point-light spheres, the structure of which could be extracted from disparity and motion as that in biological motion perception. Results showed that there was no significant difference in terms of sensitivity between the spheres rotating toward vs. away from the observers (*t (9)* = −0.04, *p*>0.1). Therefore, the perceptual asymmetry observed in Experiment 2a is more likely attributable to a mechanism specialized for processing biological motion signals, rather than an effect linked to the perception of structure from motion.

### Experiment 3: Perceptually Indistinguishable Stereoscopic Information Contributes to the Detection of Biological Motion

**Figure 3:**
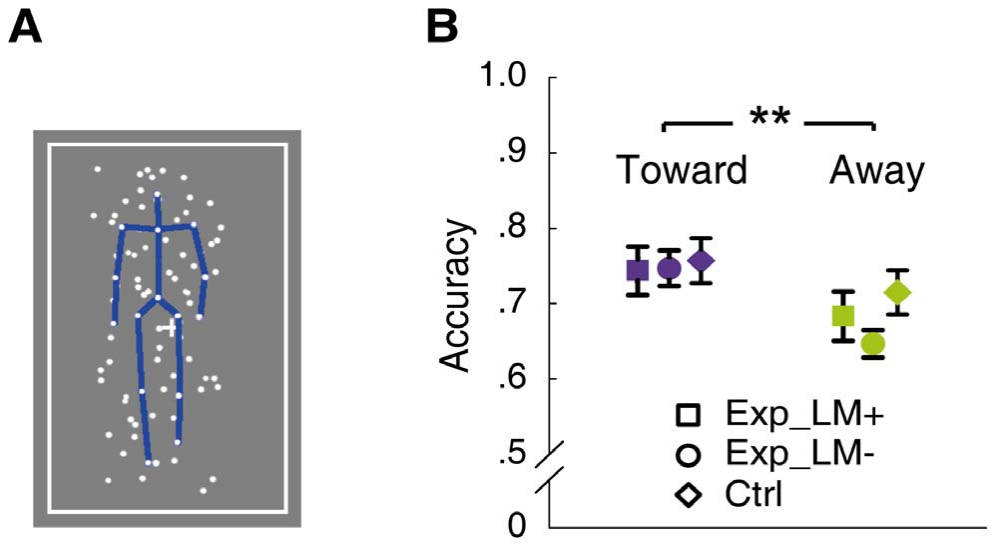
Illustration of a single frame of a sample stimulus used in the biological motion detection experiment and results of the experiment. (A) An upright point-light walker (target) embedded in dynamic noise dots. Blue lines are for illustration purpose here and were not shown in the actual experiment. (B) The accuracy of the detection task was significantly higher for the toward condition (purple) than for the away condition (green). Exp_LM+ or Exp_LM-: average performance of the experimental group, with congruent local motion (i.e., noise made from scrambled targets) or incongruent local motion (i.e., noise made from scrambled walkers rendered with disparity signals opposite to those of the target). Ctrl: average performance of the control group, with local motion conditions combined. Error bars indicate one SEM. **p<.01.

Experiment 2 demonstrated a perceptual asymmetry of biological motion perception that is related to the physical but not perceived facing orientations in depth. Because the left-right deviation discrimination task required a fine analysis of the walking direction, which probably relied on all available visual cues including depth even if they were irrelevant to the task, it is possible that the integration of the depth and other visual cues could occur merely at such fine level of 3D object processing. To examine this issue, we employed a detection task in Experiment 3 and restricted the processing of 3D biological motion to a relatively coarse level. In this task, observers were required to simply detect the presence of an upright (target) walker from dynamic noise background that masked the walker with local motion cues obtained through scrambling the point-light walkers (Figure 3a, see Methods for more details). Since observers had to concentrate on the global configuration of the human figure that emerged from figureless motion [23], [24], they were unlikely to pay close attention to the fine features of the stimuli. In addition, we manipulated the congruency of the disparity cues conveyed by the noise and the target motion, which allowed us to probe the possible influence arising from the local disparity signals on the utilization of stereoscopic information.

Results of the detection task were plotted in Figure 3b. Even under such manipulation, a robust perceptual advantage was still found for 3D walkers facing toward the observers, compared with those facing away from the observers (*F* (1, 18) = 29.96, *p*<0.001), again independent of whether the disparity-defined in-depth orientation was perceptually distinguishable or not (no interaction with participant group, *F* (1, 18) = 2.97, *p*>0.1). The overall performance was not significantly different between the two participant groups (*F* (1, 18) = 1.08, p>0.1). Moreover, local noise disparity (congruent vs. incongruent with the target) had no effect on the observers’ performance (*F* (1, 18) = 0.17, *p*>0.1), and did not interact with in-depth facing orientation (*F* (1, 18) = 0.28, *p*>0.1) or participant group (*F* (1, 18) = 0.25, *p*>0.1). Hence, it was disparity-defined 3D structure, rather than the local disparity signals, that contributed to the observed effect. Taken together, these results demonstrate that the perceptual asymmetry related to disparity-defined depth exists regardless of the perceived 3D orientation, and the perceptually indistinguishable stereoscopic information can be integrated into the visual perception of biological motion automatically at both the fine and the coarse levels.

## Discussion

Binocular disparity is well known for its importance in the visual estimation of depth. Disparity-tuned neurons have been widely found across the visual cortex and temporal and parietal areas in the primate brain [1], and several homological human brain regions are shown to correlate with the perceptual awareness of stereoscopically defined 3D information [25]–[30]. An important yet unanswered question is whether the explicit representation of stereoscopic depth is necessary for the fulfillment of its function in visual perception, which potentially involves the integration of both binocular and monocular depth cues as well as top-down mechanisms related to the specific properties of visual objects [2], [3]. The current study probed this question using a type of natural 3D objects, point-light biological motions, whose in-depth orientation information was unambiguously defined by disparity cues. In a series of experiments, we found that observers performed significantly better in detecting the presence of a stereo point-light walker or discriminating the left-right deviation of its walking direction when the walker was dichoptically displayed as walking toward compared with walking away from the observers. Remarkably, this effect persisted even when the disparity-defined 3D structure was not perceptually distinguishable owing to strong top-down influences [15], indicating an intriguing dissociation between the explicit representation of stereoscopic depth and its functional contribution to visual perception.

Previous studies have revealed that unperceived binocular depth information could participate in some involuntary functions. For instance, vergence eye movements can respond to binocular disparity cues independent of the perceived depth [31], or even in the absence of depth perception [32]. These results can be largely explained by the functional dissociation of perception and action systems, mediated by neural signals transferred through separate pathways [33]. Our results, beyond those findings, show that perceptually indistinguishable disparity-defined 3D structure may even modulate high-level visual processing, such as biological motion perception, thus highlighting a substantial role of disparity cues in visual perception independent of the observers’ depth awareness. Moreover, since the modulation took effect at both fine and coarse levels of biological motion processing and did not require explicit attention to the depth property (depth information was task irrelevant in both Experiments 2 and 3), the extraction and utilization of disparity cues in biological motion perception were rather automatic.

Our findings are in line with the literature showing that certain visual features, even not consciously perceived, can be processed and exploited by the visual system to regulate visual perception [34]–[36]. The dissociation between the perception of disparity-defined 3D structure and its functional influence on biological motion processing suggests that the extraction and utilization of binocular disparity in visual perception, like other elementary visual information, may act through mechanisms different from those that underlie its perceptual representation [34]. As a basic visual element, binocular disparity is registered as early as V1 [7], [8], whereas the perception of 3D structure from disparity requires the involvement of higher brain areas along both the dorsal and ventral cortical pathways [27]–[29]. On the other hand, the convergence of the disparity and motion cues to construct 3D objects may take place in common sites in the human brain [37], and a recent study provides solid evidence for the existence of specialized neural substrates supporting the 3D interpretation of ambiguous 2D point-light walkers by incorporating form, motion, and depth information [10]. In our study, the disparity signals, driven by early cortical responses, may be projected to brain regions where the walker’s form and motion information converge and integrate, such as the superior temporal sulcus (STS) or the extrastriate body area (EBA) [10], [38]. It is important for future work to pinpoint the exact neural mechanisms underlying the integration process and to see whether the disparity signals can directly modulate the activities of these areas, which are typically assumed to be responsible for the representation of high-level domain-specific visual information.

Notably, the perceptual asymmetry effect with respect to the in-depth motion direction has been observed only for biological rather than non-biological motion signals. What is the mechanism behind such perceptual asymmetry? Why does the effect persist even when the observers cannot explicitly discriminate the stereoscopically defined information? Since masking the local disparity cues did not influence the asymmetry regarding the in-depth orientation of biological motion (Experiment 3), local disparity signals may not directly give rise to the observed perceptual advantage of the walkers facing toward the viewers. A more probable explanation comes from the socially or biologically relevant aspects of the in-depth orientation of biological motion. Firstly, studies on biological motion perception have found that the FTV bias can be strongly modulated by observer sex and stimulus gender [12], [13], [22], indicating a potential relevance of the in-depth orientation of other people’s movement for the interpretation of social signals. Furthermore, people have greater sensitivity to approaching point-light walkers [39], and these walkers generally capture more attention and are detected faster than their depth-reversed counterparts [21], [22] but only when they are presented in upright rather than in inverted orientation [21], suggesting that the human visual system is intrinsically tuned to approaching animate signals. Although we do not negate the potential contribution from the low-level signals, the close link between the soical relevance and the 3D nature of biological motion may boost the extraction and utilization of physical depth cues in 3D biological motion perception, and lead to the perceptual asymmetry regarding the facing orientation in depth. An important implication from these findings is that, although past research on the integration of disparity and other visual cues usually employed artificial and unfamiliar shapes [40], [41], it might be more fruitful to bring in ecologically meaningful stimuli, such as biological motion, to elucidate the interplay of top-down and bottom-up factors in 3D object processing in the future.

In conclusion, the current study clearly demonstrates a robust perceptual asymmetry in response to perceptually indistinguishable stereoscopic depth information. It also points to a mechanism specialized for the visual processing of biological motion that automatically engages the 3D structure defined by binocular disparity. Together, these results suggest that the extraction and utilization of binocular depth signals can be dissociable from the conscious representation of depth in high-level 3D object perception.

## Supporting Information

Animation S1 Example of a stereoscopically presented point-light walker that is facing toward the viewer.
(GIF)

Animation S2 Example of a stereoscopically presented point-light walker that is facing away from the viewer.
(GIF)

Methods S1 (DOCX)
