# Supplementary figures and images for: Integration of 3D Structure from Disparity into Biological Motion Perception Independent of Depth Awareness
Source: PLoS One. 2014 Feb 21;9(2):e89238. doi: 10.1371/journal.pone.0089238 (PMC3931706; doi:10.1371/journal.pone.0089238)

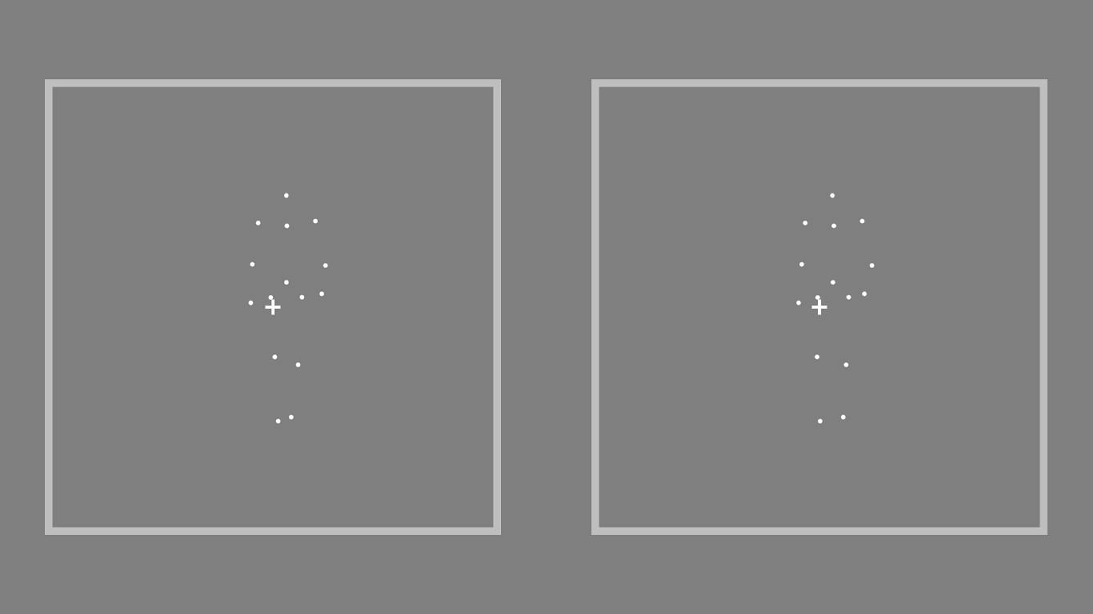

Supplement: Animation S1 — Example of a stereoscopically presented point-light walker that is facing toward the viewer. (GIF) [file pone.0089238.s001.gif]

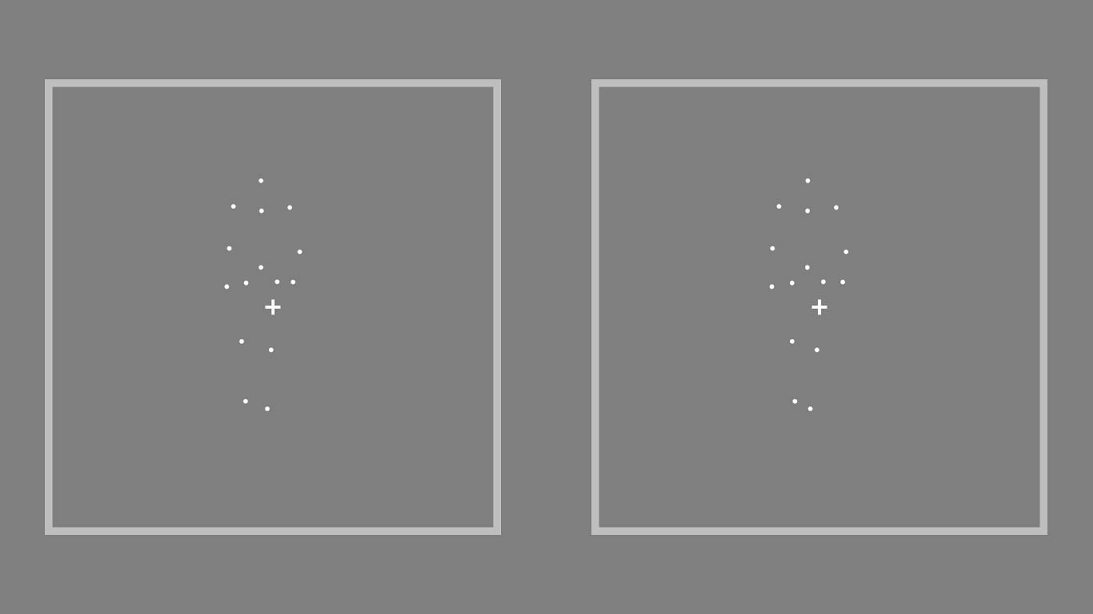

Supplement: Animation S2 — Example of a stereoscopically presented point-light walker that is facing away from the viewer. (GIF) [file pone.0089238.s002.gif]
