# Supplementary material for: Integration of 3D Structure from Disparity into Biological Motion Perception Independent of Depth Awareness
Source: PLoS One. 2014 Feb 21;9(2):e89238. doi: 10.1371/journal.pone.0089238 (PMC3931706; doi:10.1371/journal.pone.0089238)
Supplement: Methods S1 — (DOCX) [file pone.0089238.s003.docx]

**Supplemental Methods S1**

**Subject prescreening**

Note that the population distribution of depth perception of disparity-defined biological motion is continuous, although we only identified the two groups of observers in the current study for the purpose of examining the functional roles of disparity cues independent of depth awareness. The participants were prescreened informally before they took part in this study. The prescreening test adopted the same settings as that of the formal experiments. In the test, each participant reported the perceived facing orientation in depth of the stereoscopically presented point-light walkers. They were encouraged to report what they had actually perceived despite of the proportions of “toward” and “away” responses, and were allowed to practice as long as they desired. We invited the observers who always perceived the walkers as approaching and those who could correctly perceived the disparity-defined walking directions to participate in the formal experiments, though the participants did not know the criteria before they finished the prescreening test. The estimated proportions of these two groups of subjects among a sample of 48 were about 29% and 38%, respectively. Those who did not fell in the desired categories and those who reported subjective difficulty in identifying the walking direction were not enrolled in the current study (about 33%).

**Stereoscopic depth discrimination threshold test**

Ten observers (3 also participated in the main experiments) took part in the stereoscopic depth discrimination threshold test. They were identified and assigned to either the experimental group (n=5, 3 males, mean age=24.6) or the control group condition (n=5, 3 males, mean age = 25.0) according to the same criteria for the formal experiments. The experimental settings were also the same as that of the formal experiments. The stimulus was a random-dot stereogram. The observers were asked to judge whether a central square (3° × 3°), whose depth was defined by binocular disparity, was in the same plane as the background square (6° × 6°), which was always presented in the screen plane. They pressed the left or the right arrow key to indicate whether or not they perceived any depth difference between the target and the background. A 1-up/2-down staircase was adopted to measure the observers’ switch points at which the depth difference was just noticeable, i.e., the difference limen. At the beginning of each trial, the target appeared in a near or a far plane defined by a large binocular disparity (10 pixels) with equal chance. The trial ended after ten times of switches. Average value of the later 8 switch points was used as the index of depth discrimination threshold. Each observer completed a total of ten trials.
